# Gender Differences in How Family Income and Parental Education Relate to Reading Achievement in China: The Mediating Role of Parental Expectation and Parental Involvement

**DOI:** 10.3389/fpsyg.2018.00783

**Published:** 2018-05-25

**Authors:** Xiaolin Guo, Bo Lv, Huan Zhou, Chunhui Liu, Juan Liu, Kexin Jiang, Liang Luo

**Affiliations:** Collaborative Innovation Center of Assessment toward Basic Education Quality, Beijing Normal University, Beijing, China

**Keywords:** family income, parental education, reading achievement, parental expectation, parental involvement, gender differences, elementary school children

## Abstract

The impact of social economic status (SES) on children's academic outcomes has been well documented. However, the mechanisms underlying this relationship remain poorly understood. Furthermore, the process by which SES relates to academic achievement needs to be studied separately for boys and girls. Using a sample of 598 Chinese children (299 boys, 299 girls) in grades 4 to 6 and their parents, this study examined the process of how family SES, specifically family income and parental education, indirectly relates to children's reading achievement through parental expectation and parental involvement and whether this process differs between boys and girls. The results revealed that parental expectation and specific parental involvement behaviors played critical mediating roles between family SES and reading achievement. Moreover, the exact nature of these links differed by the gender of children. For boys, both the effect of parental education and the effect of family income were partially mediated by parental expectation and parent-child communication orderly. For girls, the effect of parental education was partially mediated by three separate pathways: (1) home monitoring; (2) parent-child communication; and (3) parental expectation followed by parent-child communication, while the effect of family income was fully mediated by parent-child communication. These findings suggest a process through which SES factors are related to children's academic development and identify a context under which these associations may differ. The practical implications of these findings are discussed, along with possible future research directions.

## Introduction

Social economic status (SES) is the measurement of the social status and economic status of an individual. Developmental and educational psychologists have long been interested in the impact of SES on the children's academic outcomes (Sirin, 2005; OECD, 2013). The Coleman Report argues that students' family SES is much more important in predicting academic performance than are measured differences in school resources (Coleman et al., 1966). Therefore, exploring the mechanism underlying this relationship has significance for both research and practice.

### The relationship between SES and academic achievement

A medium-to-strong relationship between SES and academic performance has been documented in several studies in different samples, such as American, African, and Asian samples (Kennedy, 1995; Ricciuti, 1999; Liu et al., 2010; Lv et al., 2016). For example, using family income and parental education to create a combined SES indicator and using the Peabody Individual Achievement test as the academic achievement indicator (which provides an assessment of achievement in five areas: mathematics, reading recognition, reading comprehension, spelling, and general information), Carlson et al. (1999) found that the relationship between SES and academic performance was *r* = 0.34 in elementary school students in grades 1–3. Gullo and Burton (1993) measured the SES of students based on whether the student participated in the federal free or reduced-price lunch program and found that the relationship between SES and the mean of reading and math was *r* = 0.12. A meta-analysis of 101 articles found that the relationship between SES and reading was *r* = 0.307, and the relationship between SES and math was *r* = 0.246 (White, 1982). In addition, in a more recent meta-analysis of 201 studies, the relationship between SES and reading was *r* = 0.32, and the relationship between SES and math was *r* = 0.35 (Sirin, 2005). This relationship has also been found in China. According to the Program for International Student Assessment (PISA) 2012 results, 15.1% of differences in mathematics performance and 15.6% of differences in reading performance among Chinese students (from Shanghai) were explained by disparities in students' SES, approximately the same as Organization for Economic Co-operation and Development (OECD) average levels, 14.8 and 13.1%, respectively.

### The mediating role of parental involvement in the relationship of SES and academic achievement

It is noteworthy that the impact of family SES on academic achievement includes not only direct effects but also indirect effects (Conger and Donnellan, 2007). According to the family stress models (Elder and Caspi, 1988; McLoyd, 1989), families' economic hardship influences children's developmental outcomes indirectly through a series of mediating family processes. Specifically, economic difficulties lead to economic pressure in the family and parental increased risk for emotional distress, and this, in turn, results in poor parental involvement (Conger and Donnellan, 2007). Parental involvement generally refers to parents' participation in their children's school education through communication with school personnel, through discussions about school-related topics with children, through attendance at school activities, and through the cultivation of child behaviors that promote educational success (Jenkins, 1997; Hill and Taylor, 2004; Hill and Tyson, 2009). The positive effect of parental involvement on children's academic development has been found in different cultures (Cheung and Pomerantz, 2011; Castro et al., 2015). A recent meta-analysis of 37 studies in kindergarten, primary and secondary schools showed that the effect size of the association between parental involvement and student academic achievement was *r* = 0.124 (Castro et al., 2015). A cross-cultural study reported that parents' increased involvement predicted children's improved achievement similarly in China and the United States (Cheung and Pomerantz, 2011). Furthermore, a relationship between family SES factors and parental involvement has been found (Sui-Chu and Willms, 1996; Englund et al., 2004). Wei et al. (2015) observed that Chinese parents with higher educational attainment were more involved in their children's school than parents with lower educational attainment. Camacho-Thompson et al. (2016) also found that low-income parents are typically less involved with their children than affluent parents.

To our knowledge, only three studies have examined whether the influence of SES on children's academic achievement was mediated by parental involvement. Based on National Educational Longitudinal Study of 1988 data, Altschul (2012) investigated the effects of multiple SES components on the academic achievement of Mexican-American youth. The results showed that SES was predictive of children's academic achievement, and parent involvement mediated the influence of both family income and maternal education on youth's academic achievement in Tenth grade. However, with Ghanian youth as participants, Chowa et al. (2013) were unable to replicate the findings of Altschul (2012). In their study, parent SES was not an effective predictor of parental involvement in Ghana (Chowa et al., 2013). In a study of kindergarten children, Cooper et al. (2010) concluded that parental involvement did partially mediate the association between family poverty and children's reading and math achievement; however, the mediation model was not equivalent across race (Asian, Black, Hispanic, and White).

### The mediating role of parental expectation in the relationship of SES and academic achievement

Parental expectation, which is defined as realistic beliefs that parents have about their children's future achievement (Yamamoto and Holloway, 2010), has been found to be fundamental to children's academic success. Three meta-analysis studies found that parental expectation has the strongest relationship to student academic outcomes compared with other parental beliefs and behaviors (Fan and Chen, 2001; Wilder, 2014; Castro et al., 2015). Furthermore, SES, especially parental education, has been identified as a strong predictor of parental expectation. Parents with backgrounds of moderate-to-high income and education usually have higher expectation for their children's academic achievement than parents with low-SES backgrounds (Gill and Reynolds, 1999; Zhan, 2006).

The results of the correlation and hierarchical regression analysis in previous studies have provided indirect evidence for the mediating role of parental expectation. On one hand, it has been found that SES, parental expectation and children's academic outcomes were related to each other (Englund et al., 2004; Froiland et al., 2012; Wang, 2015). On the other hand, there was always a marked reduction in the associations between parental education or family income and children's academic performance, after controlling for parental expectation (Zhan, 2006; Froiland et al., 2012; Wang, 2015). These studies suggested that parental expectation might account for part of the effect of SES on children's outcomes.

### The moderating role of child's gender

Though family SES has been confirmed to be stably associated with children's educational outcomes, some researchers suggest that the magnitude of this association might vary by the gender of children (Dubow et al., 2009). In one of the few studies examining this question, Autor et al. (2015) found that boys who came from socioeconomically disadvantaged households had lower achievement scores and lower high-school completion rates than their sisters. The author suggested that one explanation might be that parental investments differed between boys and girls according to family disadvantage. For instance, parents in low-SES families usually spend more time mentoring and interacting with girls than boys (Bertrand and Pan, 2013; Baker and Milligan, 2016). Moreover, other studies have reported that even the same parenting behaviors could affect academic achievement differently, depending on the gender of children (Stage and Hossler, 1989; Brown et al., 2009). From a cognitive developmental perspective, Huston (1983) suggested that boys and girls seem to interpret the environment around them through a *gender filter*, paying attention to different things in the environment or valuing the same things but for different reasons, depending on their own gender (Linver and Silverberg, 1997). For example, as autonomy is traditionally regarded as a male quality, boys may consider this facet of parenting more important than girls and in turn boys' academic achievement may be impacted more strongly by autonomy-supporting behavior from parents than that of girls. Findings on the effect of gender in the relationship between parenting and children's academic achievement have supported this theory. Tam (2009) found that Chinese mothers' academic efficacy, which referred to parents' perceptions of their own competency in helping children to cope with school work, had a stronger positive effect on academic performance for primary school-age boys than for girls, while mothers' psychological control had a negative effect for boys and no effect for girls. Fulton and Turner (2008) found positive associations between parental autonomy granting and college students' GPA only in males. These findings raise another question of whether the process linking family SES with academic achievement differs by gender as well.

Although these studies have improved our understanding of the mechanism by which SES relates to children's academic achievement, four important issues still remain unaddressed. First, despite many studies have provided indirect evidence for the mediating role of parental expectation, we know of no studies that have specifically studied and discussed the mediating role of parental expectation in relations between family SES matters and children's achievement directly. Second, developmental niche theory has suggested that parenting behaviors are also shaped by parent's cultural beliefs about parenting goals and children's development (Super and Harkness, 1986). Based on this theory, a series of studies have found evidence of a positive association between parental expectation and parental involvement. Yamamoto and Holloway (2010) suggested that fostering greater parental involvement was one of the pathways by which parental expectations were thought to affect student achievement. A similar point has also been made by Halle et al. (1997) and Seyfried and Chung (2002). This link between parental expectation and parental involvement may further provide insight into the process by which expectation impacts children's academic achievement (Seginer, 1983; Seyfried and Chung, 2002). However, no previous studies, to our knowledge, have explored the mediating effect of both parental expectation and parental involvement on relationship between SES and academic achievement. Third, although some researchers suggest that the magnitude of this association might vary by the gender of children, how this proposed effect occurs has remained unaddressed. Given this potentially moderating role of gender, the current study also examined whether the mediating model differed between boys and girls. Fourth, most previous studies did not distinguish different subject achievements as the children's academic achievement indicator. However, the meta-analysis indicated that single subject achievement measures yielded significantly larger correlations with SES than general achievement measures (Sirin, 2005). Thus, in this study, we specifically focused on children's reading achievement, not only because reading achievement is a prerequisite for all other school success (Farkas, 1996), but also because it has been suggested that distinct mediating mechanisms operate in the association between family SES and different academic outcomes (Eamon, 2002), and reading achievement has been reported to be more strongly affected by family factors than other subject areas, such as mathematics (Mercy and Steelman, 1982; Marjoribanks, 1989).

### The current study

**Figure 1:**
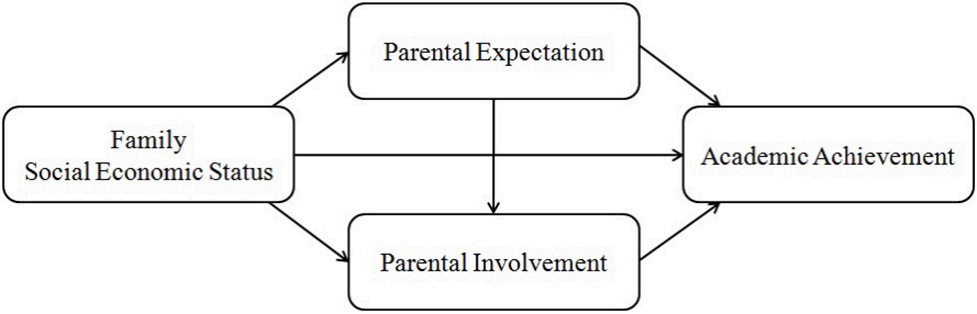
The hypothesized conceptual model.

The main goal of the present study was to test the hypothesized model of the impact of family SES on reading achievement among Chinese students through parental expectation and involvement and to examine the gender differences in this process (see Figure 1). Considering that various components of SES may have different effects on children's development (Sirin, 2005; Conger and Donnellan, 2007), family SES in this study was measured by family income and parental education, and their unique contributions to reading achievement were examined. Given that the relationship between the evaluation of occupational prestige and levels of economic development is apparently more complex in China than it is in Western societies (Hodge et al., 1966) and that the consistency evaluation of occupational prestige in China is much lower than in other countries (Li, 2000), parental occupation was not included in the current study. Based on the literature review, our hypotheses were as follows: (a) both parental education and family income influenced reading achievement among Chinese students; and (b) these effects were mediated by parental expectation and some types of parental involvement, after controlling for children's age and sibling status. It was expected that the child's gender may affect these predictive relations, but the lack of empirical evidence prevented us from making direct hypotheses about gender effects.

## Materials and methods

### Ethical statement

All procedures in this study were approved by the Institutional Review Board of the Collaborative Innovation Center of Assessment toward Basic Education Quality, Beijing Normal University. Written informed consent to participate in the study was obtained from parents of all child participants before evaluation.

### Participants and procedure

The participants were 624 students in grades 4–6 and their parents, who were recruited from two elementary schools in Liaocheng, a typical medium-sized city in China. The per capita annual disposable income of Liaocheng was 18,085 Chinese yuan/person in 2013, an amount close to the national average (18,310.8 Chinese yuan/person). The average student-teacher ratio in Liaocheng in 2013 was 17.96, similar to the national average ratio of 16.76 (Bureau of Statistics of Shandong Province., 2014; National Bureau of Statistics of China, 2014). Two classes in each grade were randomly selected from each school to participate in this project. After agreeing to participate, the primary caregivers of these students completed questionnaires regarding their expectation for children's educational attainment, their involvement in children's educational activities, and some demographic information, including the child's age, gender, sibling status, family income, and mothers' and fathers' educational attainment.

After excluding 26 incomplete responses (lack of parental response), the final samples for this study included 598 (299 boys and 299 girls) students and their parents (207 fathers and 391 mothers). The proportions of each grade were 30.3, 36.1, and 33.6%, respectively. The mean ages of the students and parents were 10.92 years (*SD* = 0.94, range = 9–14) and 37.68 years (*SD* = 2.70, range = 28–49), respectively. Because of China's One-Child Policy, 64% (*N* = 382) of the students were only children.

### Measures

#### Parent and family characteristics

For this study, parental education and family income were used to characterize family SES. Parental education was based on the child's primary caregiver's response concerning his/her own and his/her spouse's highest educational attainment. The possible educational categories based upon the Chinese educational system were as follows: 1 = primary school or below (2% for fathers, 4.7% for mothers); 2 = middle school (21.4% for fathers, 25.3% for mothers); 3 = high school (24.1% for fathers, 26.6% for mothers); 4 = junior college degree (24.6% for fathers, 19.9% for mothers); 5 = bachelor degree (25% for fathers, 21.4% for mothers); 6 = master's degree or above (2.9% for fathers, 2.2% for mothers). The correlation between fathers' and mothers' education was *r* = 0.75, *p* < 0.01. And fathers' education (*M* = 3.58, *SD* = 1.21) was slightly higher than mothers' education (*M* = 3.35, *SD* = 1.26), *t*_(597)_ = 6.30, *p* < 0.01, *d* = 0.52. To obtain the most accurate picture of the education available in the household, the highest education in the household was used as the indicator of family education. According to the latest Sixth National Population Census of the People's Republic of China in 2010, the education level distribution of the urban economically active population was as follows: 8.7% completed primary school or below, 39.5% completed middle school, 25.7% completed high school, 14.3% completed junior college, 10.5% had a bachelor degree, and 1.3% had a master's degree or above, which means that our sample had a slightly higher educational level than a truly representative one, but the discrepancy was small.

Family income represented the total combined family income during the last year for all members of the family, which was reported in the following categories: 1 ≤ ¥3,600 (4.8%); 2 = ¥3,601–7,200 (10.0%); 3 = ¥7,201–14,000 (8.4%); 4 = ¥14,001–30,000 (16.2%); 5 = ¥30,001–50,000 (28.1%); 6 = ¥50,001–100,000 (28.8%); 7 > ¥100,000 (3.7%). Since the average family population was 3.09 people in China, and the per capita annual disposable income was 18,310.8 Chinese yuan/person (National Bureau of Statistics of China, 2014), the average yearly family income in China is approximately 56,580 Chinese yuan, which is roughly in line with our data.

#### Parental expectation

Parental expectation for achievement was measured by the child's primary caregiver's response to the question, “How much schooling do you expect that your child will complete?” Measurements ranged from 1 = graduate from middle school, to 5 = beyond a master's degree (Seginer, 1983).

#### Parental involvement

The Parental Involvement Questionnaire (parent report) (Wu et al., 2013) is a 29-item self-report inventory adapted from previous parental involvement research (Walker et al., 2005; Green and Hoover-Dempsey, 2007; Green et al., 2007). All items were developed based on Chinese culture. The items describing parents' involvement in their children's educational activities inside/outside school are rated on a 4-point scale ranging from never (1) to always (4). This questionnaire contains five sub-scales representing five important and well-recognized dimensions of involvement: (a) parent-school contact: parents were asked to indicate how often they visited the school, attended school events (parent meetings, performances, athletic, and extracurricular activities) and stayed in contact with teachers and school personnel; (b) parent-child communication: parents were asked to indicate how often they conversed with their children about learning-related topics and shared school experiences, e.g., discussing school activities, academic performance, showing interest in children's progress at school or things the child is interested in, or discussing the value of a good education; (c) learning assistance: parents were asked to indicate how often they helped their children complete homework and prepare for coming examinations; (d) parent-child activity: parents were asked how frequently they spent time with their elementary school children in extracurricular activities, such as visiting museums; and (e) home monitoring: parents were asked to report how frequently they monitor children's use of time, e.g., setting limits on TV watching and establishing a daily family routine.

Item-level confirmatory factor analyses were conducted to confirm the structural dimensions of the Parental Involvement Questionnaire in this study. The five-factor model yielded an acceptable fit: χ^2^ = 825.452, *df* = 367, *p* < 0.001, χ^2^/*df* = 2.249, *SRMR* = 0.053, *CFI* = 0.912, *TLI* = 0.902, *RMSEA* = 0.046. The Cronbach's alphas for the five sub-scales and the entire questionnaire ranged from 0.48 to 0.91.

#### Reading achievement

Children's mid-term and final exam grades in Chinese were used as indicators of reading achievement. The mid-term and final exam were designed by a group of experienced teachers and were organized by school themselves. The content of the mid-term exam differed from that of the final exam and exam content was different for each school and for each grade. However, all of the exams were based on curriculum standards developed by Ministry of Education of the People's Republic of China. In China, the grades were originally numerical, ranging from 0 to 100. Grades were standardized within the school and grade to incorporate differences among the grading systems for each school and grade (Cheung and Pomerantz, 2011). The correlation between the mid-term and final exam grades was *r* = 0.74, *p* < 0.01. Scores of the mid-term and final exam grades were averaged to form a single index of reading achievement; higher numbers reflect higher levels of reading achievement.

### Analytic approaches

The statistical analyses were performed by SPSS version 19 and Amos version 20. First, we conducted a bivariate correlation to assess the strengths of linear relationships among all variables. We then established the hypothesized model. In this model, parental education, family income, parental expectation and reading achievement were observed variables, while five dimensions of parental involvement were latent variables. For the five dimensions of parental involvement, parcels of items were used as the manifest variables per dimension rather than single items. Parcels were built according to the item-to-construct balance technique (Little et al., 2002). Specifically, for each of the five dimensions, the two items with the highest loadings were set as anchors of the respective parcels, and the two items with the lowest loadings were then added to the parcels in inverted order, resulting in two parcels per latent variable in the model.

To determine whether the overall model was similar or different between boys and girls, multiple group comparisons were performed. In this analysis, boys and girls were treated as two subgroups, and two models were estimated. The unconstrained model allowed the structural paths to vary between boys and girls. The constrained model constrained all parameter estimates for boys and girls to be equal. If the constrained model resulted in a statistically significant decrement of model fit in comparison to the unconstrained model, the models are not equivalent for the two groups (Arbuckle, 2011). Once the difference between boys and girls was confirmed, SEM analyses were conducted separately for each gender.

We employed several criteria to assess the fit for our model, including χ^2^, which is ideally non-significant, indicating a good fit of the model. However, the value of χ^2^ was sensitive to large sample sizes (Marsh and Balla, 1994). Thus, other statistics were also used to assess the model fit, including the Tucker-Lewis index (*TLI*), comparative fit index (*CFI*), and the root mean squared error of approximation (*RMSEA*). A cut-off value of the *TLI* and *CFI* should be 0.90 or greater, indicating a close fit (Hox and Bechger, 1998). The value of *RMSEA* should be approximately 0.05, representing a close fit (Brown and Cudeck, 1992).

Finally, a bootstrapping analysis was conducted to test mediating effects (Preacher and Hayes, 2008). The bias-corrected bootstrap method with 1,000 resamples was used to calculate the 95% confidence intervals (CI). The effect was statistically significant if the CI did not include zero.

## Results

### Descriptive and correlational statistics

Table 1 provides means and standard deviations on all of the assessed variables for the total sample and for boys and girls separately. Differences between boys and girls emerged only for parent-school contact, *t*_(596)_ = 2.94, *p* < 0.01, *d* = 0.24, and reading achievement, *t*_(596)_ = −5.32, *p* < 0.01, *d* = 0.44.

**Table 1:** Descriptive statistics and *t*-tests for all variables by gender.

**Variable**	***M*** **(*****SD*****)**			***t*-Test**
	**Boys**	**Girls**	**Total**	
Parental education	3.77 (1.25)	3.70 (1.19)	3.74 (1.22)	0.74
Family income	4.51 (1.60)	4.57 (1.49)	4.54 (1.55)	−0.50
Parental expectation	4.59 (0.55)	4.64 (0.49)	4.62 (0.52)	−1.17
Home monitoring	3.38 (0.47)	3.32 (0.49)	3.35 (0.48)	1.45
Learning assistance	3.16 (0.57)	3.14 (0.60)	3.15 (0.58)	0.30
Parent-child communication	3.41 (0.42)	3.40 (0.43)	3.40 (0.42)	0.32
Parent-child activity	2.49 (0.58)	2.42 (0.59)	2.45 (0.59)	1.42
Parent-school contact	2.60 (0.62)	2.45 (0.62)	2.53 (0.62)	2.94**
Reading achievement	-0.20 (1.05)	0.20 (0.74)	0.00 (0.78)	−5.32**

** *p < 0.01*.

Prior to conducting structural equation modeling analyses, simple correlations were examined. Table 2 shows the Pearson correlations among all variables separately for boys and girls, and the results showed that the majority of correlations were in the expected directions.

**Table 2:** Correlation matrices for study variables by gender.

	**1**	**2**	**3**	**4**	**5**	**6**	**7**	**8**	**9**	**10**	**11**
1.Child's age	—	−0.17**	−0.12*	−0.02	−0.01	−0.02	−0.13*	−0.11	−0.06	−0.02	−0.05
2.Child's sibling status	−0.11*	—	0.40**	0.06	0.09	0.07	0.15*	0.19**	0.27**	0.08	0.06
3.Parental education	−0.18**	0.47**	—	0.14*	0.18**	0.13*	0.17**	0.24**	0.32**	0.20**	0.23**
4.Family income	−0.07	0.16**	0.26**	—	0.14*	0.02	0.04	0.17**	0.08	0.03	0.18**
5.Parental expectation	−0.13*	0.23**	0.36**	0.19**	—	−0.05	0.07	0.17**	0.17**	0.11	0.17**
6. Home monitoring	−0.04	0.13*	0.09	0.03	0.05	—	0.45**	0.37**	0.27**	0.32**	−0.09
7.Learning assistance	−0.01	0.14*	0.10	0.02	0.04	0.38**	—	0.35**	0.38**	0.37**	0.00
8.Parent-child communication	−0.14*	0.23**	0.23**	0.13*	0.27**	0.38**	0.39**	—	0.50**	0.39**	0.16**
9.Parent-child activity	−0.12*	0.06	0.24**	0.02	0.17**	0.33**	0.36**	0.51**	—	0.63**	0.05
10.Parent-school contact	0.03	0.06	0.13*	−0.05	0.14*	0.34**	0.46**	0.44**	0.54**	—	−0.01
11.Reading achievement	−0.15**	0.27**	0.30**	0.26**	0.24**	0.04	−0.01	0.27**	0.18**	0.04	—

Girls are above the diagonal, boys are below the diagonal.

Child's sibling status: 0 = not only child, 1 = only child.

* p < 0.05;

** *p < 0.01*.

For both genders, parental education, family income, and parental expectation were all significantly and modestly to moderately related to reading achievement, and both parental education and family income were positively related to parental expectation. The correlation pattern between five dimensions of parental involvement and other assessed variables, however, was not the same for boys and girls. Parent-child communication and parent-child activity reported by boys' parents were positively related to boys' reading achievement. For girls, only parent-child communication was significant. Parental education was significantly correlated with three dimensions of parental involvement—parent-child communication, parent-child activity and parent-school contact—for boys, and parental education was correlated with all five dimensions for girls. Parental expectation was positively related to parent-child communication and parent-child activity for both boys and girls and positively related to parent-school contact for boys only. A positive relation was found between family income and parent-child communication for both genders. These correlations provide some initial evidence that the process of how family SES relates to children's reading achievement through parental expectation and involvement is not the same for boys and girls.

Finally, statistically significant correlations were found between child characteristics (age and sibling status) and other assessed variables; thus, child's age and sibling status were used as control variables in the next analysis.

### Multiple-group comparison of gender differences

**Figure 2:**
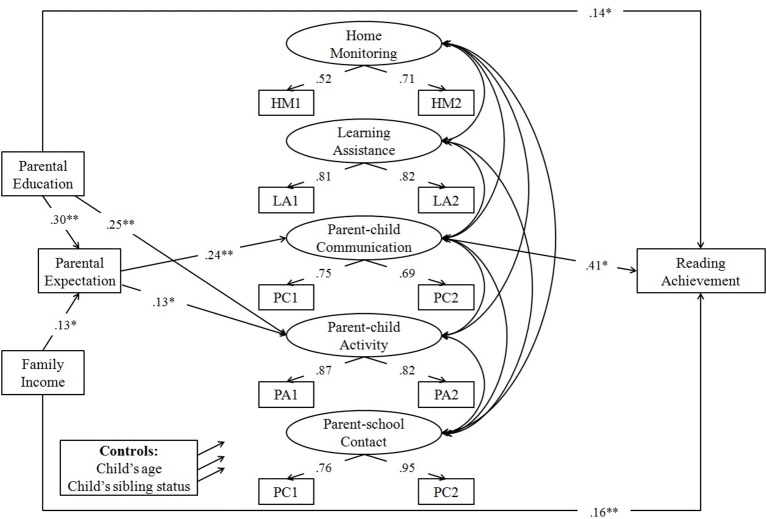
Model predicting reading achievement with parental expectation and parental involvement as potential mediators: Boys (*N* = 299). *p < 0.05; **p < 0.01.

**Figure 3:**
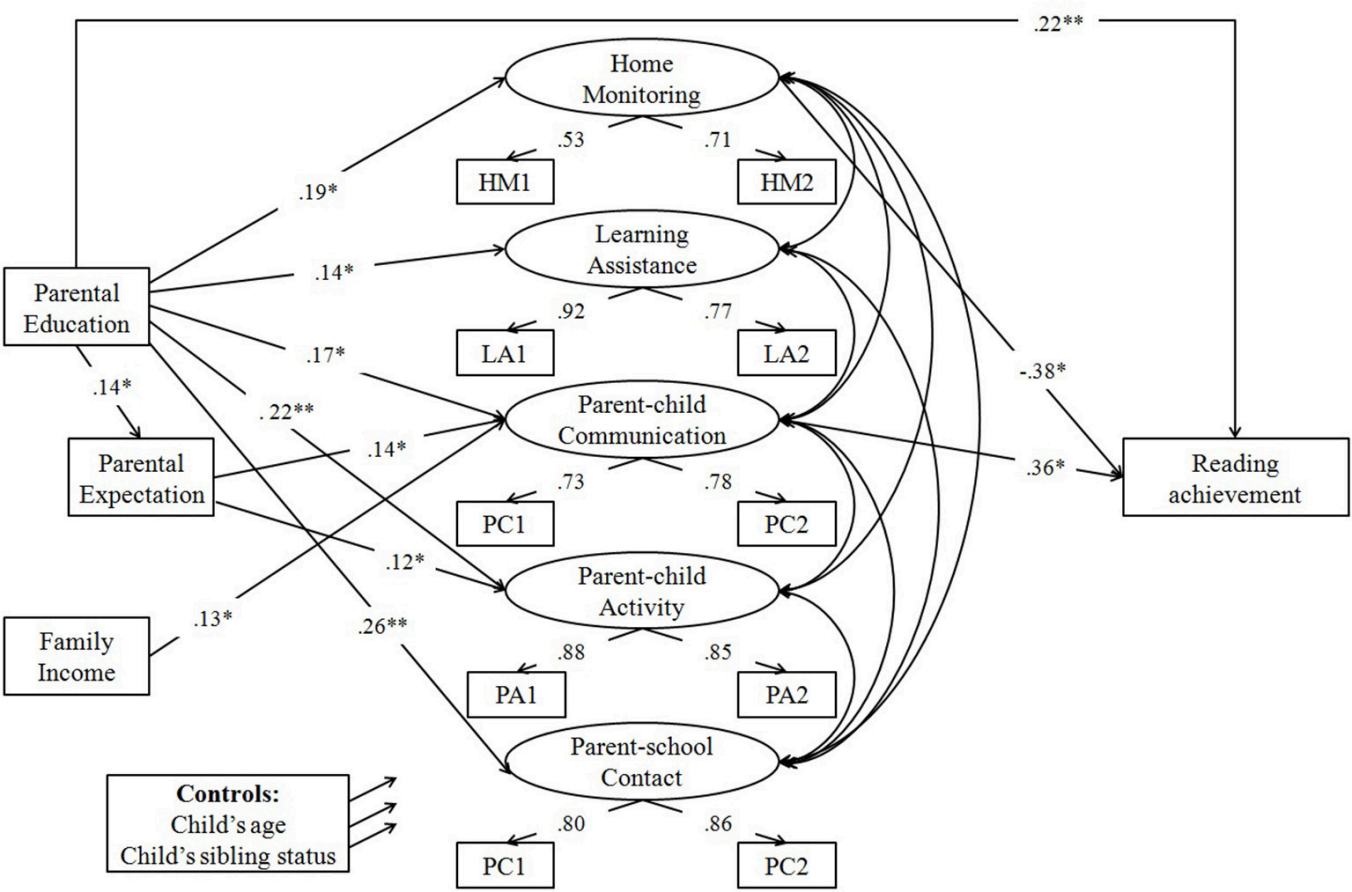
Model predicting reading achievement with parental expectation and parental involvement as potential mediators: Girls (*N* = 299). *p < 0.05; **p < 0.01.

To examine whether there was gender difference in the process by which family SES relates to reading achievement, the hypothesized model was constructed, and multiple group comparisons were conducted. The results showed that both the unconstrained model and the constrained model fit the data well (see Table 3). However, the chi-square difference test was significant, Δχ^2^ = 68.23, *df* = 44, *p* = 0.01, suggesting that the process of how family SES relates to reading achievement was not the same for the two groups. Thus, SEM analyses were conducted separately for each gender. The results of these analyses appear in Figures 2, 3. For simplicity, only significant standardized path coefficients were shown, but as dictated by the hypothesized model, all direct and indirect paths were tested.

**Table 3:** Fit indices for mediation model with parental expectation and involvement as mediators.

**Model**	**χ^2^**	***df***	***p***	***CFI***	***TLI***	***RMSEA***
Unconstrained	194.668	120	0.000	0.973	0.946	0.032
Constrained	262.897	164	0.000	0.964	0.947	0.032
M boys	95.990	60	0.002	0.974	0.948	0.045
M girls	98.678	60	0.001	0.972	0.943	0.047

### Structural models for each gender

For boys, the model was a good fit for the data (see Table 3), and a large percentage of the variance was explained (*R*^2^ = 0.63). As can been seen in Figure 2, parental education significantly predicted parental expectation, parent-child activity and reading achievement; family income significantly predicted parental expectation and reading achievement; parental expectation significantly predicted parent-child communication and parent-child activity; and parent-child communication significantly predicted reading achievement.

Table 4 showed the results of the mediating effect test of parental education on reading achievement. As seen in Table 4, the indirect effect of parental expectation followed by parent-child communication in the link between parental education and reading achievement was significant. In addition, a significant total effect and a significant direct effect were observed, which suggested that this indirect pathway partially mediated the effect of parental education on reading achievement.

**Table 4:** Direct, indirect and total effects of parental education on reading achievement.

**Model pathways**	**Boys**			**Girls**		
	**Estimated** **effect**	**95% CI**		**Estimated** **effect**	**95% CI**	
	**Lower**	**upper**	**Lower**	**upper**
**DIRECT EFFECT**						
PE → RA	0.118*	0.017	0.232	0.137**	0.046	0.259
**INDIRECT EFFECTS**						
PE → HM → RA	−0.008	−0.085	0.019	−0.045*	−0.237	−0.002
PE → LA → RA	−0.006	−0.054	0.010	0.012	−0.012	0.085
PE → PCC → RA	0.031	−0.013	0.157	0.037*	0.001	0.168
PE → PCA → RA	0.013	−0.027	0.075	−0.030	−0.151	0.037
PE → PS → RA	−0.009	−0.081	0.012	0.005	−0.042	0.069
PE → PEX → RA	0.001	−0.045	0.040	0.004	−0.021	0.024
PE → PEX → HM → RA	0	−0.015	0.015	0.003	−0.002	0.020
PE → PEX → LA → RA	0	−0.007	0.009	0.001	−0.001	0.008
PE → PEX → PCC → RA	0.025**	0.006	0.085	0.005*	0.001	0.021
PE → PEX → PCA → RA	0.002	−0.002	0.017	−0.002	−0.014	0.003
PE → PEX → PS → RA	−0.003	−0.021	0.004	0	−0.004	0.007
Total effect	0.164**	0.068	0.263	0.126**	0.054	0.201

PE, parental education; FI, family income; PEX, parental expectation; HM, home monitoring; LA, learning assistance; PCC, parent-child communication; PCA, parent-child activity; PS, parent-school contact; RA, reading achievement.

* p < 0.05;

** *p < 0.01*.

Table 5 showed the results of the mediating effect test of family income on reading achievement. As seen in Table 5, the indirect effect of parental expectation followed by parent-child communication in the link between family income and reading achievement was significant. In addition, a significant total effect and a significant direct effect were observed, which suggested that this indirect pathway partially mediated the effect of family income on reading achievement.

**Table 5:** Direct, indirect and total effects of family income on reading achievement.

**Model pathways**	**Boys**			**Girls**		
	**Estimated** **effect**	**95% CI**		**Estimated** **effect**	**95% CI**	
	**Lower**	**upper**	**Lower**	**upper**
**DIRECT EFFECT**						
FI → RA	0.101*	0.018	0.184	0.038	−0.055	0.093
**INDIRECT EFFECTS**						
FI → HM → RA	−0.001	−0.040	0.025	−0.005	−0.062	0.062
FI → LA → RA	0	−0.017	0.020	0	−0.021	0.018
FI → PCC → RA	0.023	−0.009	0.116	0.023*	0.002	0.098
FI → PCA → RA	−0.001	−0.026	0.004	0	−0.02	0.025
FI → PS → RA	0.005	−0.007	0.047	−0.001	−0.016	0.017
FI → PEX → RA	0.001	−0.018	0.015	0.002	−0.017	0.015
FI → PEX → HM → RA	0	−0.006	0.005	0.002	−0.002	0.014
FI → PEX → LA → RA	0	−0.002	0.003	0	−0.001	0.005
FI → PEX → PCC → RA	0.008*	0.001	0.037	0.003	−0.001	0.013
FI → PEX → PCA → RA	0.001	−0.001	0.008	−0.001	−0.008	0.001
FI → PEX → PS → RA	−0.001	−0.010	0.001	0	−0.002	0.004
Total effect	0.137**	0.056	0.226	0.061**	0.012	0.117

* p < 0.05;

** *p < 0.01*.

For girls, the model was also a good fit for the data (see Table 3), and a large percentage of the variance was explained (*R*^2^ = 0.47). As can been seen in Figure 3, parental education significantly predicted parental expectation, all five dimensions of parental involvement and reading achievement; family income only predicted reading achievement; parental expectation significantly predicted parent-child communication and parent-child activity; and home monitoring and parent-child communication significantly predicted reading achievement.

As seen in Table 4, similar to the boys' model, the indirect effect of parental expectation followed by parent-child communication in the link between parental education and reading achievement was significant. Moreover, two additional significant indirect effects were also found: the indirect effect through home monitoring and the indirect effect through parent-child communication. In addition, a significant total effect and a significant direct effect were observed, which suggested that these three indirect pathways partially mediated the effect of parental education on reading achievement.

As seen in Table 5, only the indirect effect of parent-child communication in the link between family income and reading achievement was significant. In addition, a significant total effect but a non-significant direct effect were observed, which suggested that this indirect pathway fully mediated the effect of family income on reading achievement.

## Discussion

The study proposed a model to examine the process of how family SES, specifically parental education and family income, relates to reading achievement among Chinese students through parental expectation and parental involvement and to examine whether this process differs between boys and girls. The study results showed that in Chinese families, parental education and family income were moderately correlated with children's reading achievement for both boys and girls, which was consistent with the results of Sirin (2005), who reported that the SES–achievement correlation was *r* = 0.29 for family income, and *r* = 0.30 for parental education. The data also supported parental expectation and parental involvement as mediators of the relationship between family SES and achievement. More interesting was the finding that the exact nature of the indirect process of how family SES related to reading achievement differed for the two gender groups.

For boys, parental education was related to children's reading achievement both directly and indirectly, as was family income. These findings demonstrated that the association between family SES and reading achievement was partially explained by the indirect paths included in the model. The data in this study further revealed that parental education and family income were related to reading achievement via the same mechanism: these two SES components all had a positive relationship to parental expectation, which, in turn, maintain a predictive relation with parent-child communication. Meanwhile, parent-child communication continued to show a direct relationship with reading achievement. This result was consistent with previous studies that have found a significant relationship between SES and parental expectation (Halle et al., 1997) and a significant relationship between parental expectation and parental involvement (Yamamoto and Holloway, 2010). Our findings expand the family stress models (Elder and Caspi, 1988; McLoyd, 1989) and indicated that the SES may affect parents' belief (e.g., parental expectation) first, which in turn, influence parental involvement. Even though parental education and parental expectation positively predicted parent-child activity, the activity had no influence on reading achievement. This finding is in line with existing evidence that after controlling for family SES and parental expectation, parents' participation with children in play activities was not related to the academic achievement of children 8–12 years of age in African American and European American families (Davis-Kean, 2005). We share the concern of the author that this finding is possible due to the age of the children in the study. As children enter middle childhood, parent-child activity is perhaps no longer the principal form of home-based involvement, which can be seen from the means of the dimensions of parental involvement in Table 1, and is perhaps more likely to improve the relationship between parents and children than to improve children's achievement.

For girls, the process is different and more complex than for boys. Family income only has an indirect effect on reading achievement, while parental education has both a direct and indirect effect on achievement. The results for the SEM model suggest that the effect of family income on children's reading achievement was almost entirely explained by parental involvement factors; however, the effect of parental education was partially mediated by parental expectation and involvement. Family income was not related to parental expectation but did have a direct relationship with parent-child communication, which in turn had a moderate relationship to children's achievement. These results indicated that parental belief was not an effective mediator in the relationship between family income and girls' reading achievement. It is possible that a higher level of family income indicates more family wealth, and the more family wealth parents accumulate, the higher the expectation for the next generation to have sufficient ability to keep and increase the family wealth held by the parents. Given the tradition of a preference for sons in China, a male is the primary successor to the family property and business, not just because of parents' dependence on sons for support in old age, but because daughters are considered to be lost to their natal family when they get married to another family (Banister, 2004; Ikels, 2004). Thus, higher family income predicted higher parental expectation for future educational attainment among boys but not girls. Parental education, in contrast, did have a significant relation with parental expectation for girls, and parental expectation was in turn related to parent-child communication, which continued to have a relation with children's achievement. Moreover, parental education also had directly positive relations with all five dimensions of parental involvement, but only home monitoring and parent-child communication were finally related to reading achievement. It should be noted that the effect of home monitoring on children's reading achievement was negative, which was consistent with the findings of McNeal's study (McNeal, 2001), but contrary to the findings of Fan and Chen's study (Fan and Chen, 2001). It seems probable that too much intervention in children's learning, especially when the children are in higher grades, might hinder the development of the independent learning and thinking skills that are clearly associated with children's learning outcomes (Karreman et al., 2006). In addition, in light of girls' high self-control abilities, girls may see parents' monitoring as more intrusive than boys. It is also likely that parent's increased supervision is a reaction to the children's low academic achievement. Certain aspects of home monitoring, such as set rules regarding leisure and homework time, might be prompted by children's poor performance in school (Wilder, 2014).

Taken together, despite the processes of the two groups were somewhat different, the findings provided support for the mediation hypothesis that the effects of family SES on children's reading achievement are mediated by both parental expectation and parental involvement. Of particular note is the same relational patterns between boys and girls in the relationships among parental expectation, parental involvement and children's reading achievement. It appears that parents' expectation was positively related to two components of parental involvement, and one of them, parent-child communication, fully mediated the relationship between parental expectation and children's achievement when family SES was controlled for in analyses.

This result revealed that the day-to-day conversations between parents and children about school-related activities seem to be the most effective form of parental involvement that improved children's academic achievement in China. This finding was consistent with those of previous research, which reported that parent-child communication was the most important factor in promoting Chinese students' learning among five types of parental practices (Wei, 2012). Through the communication channel, parents convey to the child the importance of school and education and let their child know how much he or she is expected to achieve. Parent-child communication also provides an opportunity to strengthen connections and maintain relationships between parents and children, which can facilitate children's perceptions and acceptations of parental expectations (Albert and Ferring, 2012; Wu et al., 2018). In addition, successful communication improves parents' ability to adjust the home environment and their own parenting practices to meet the needs of their child as they receive information about the performance of their child in school and the requirements of the school. However, the Chinese traditional hierarchical relationship within the family limits explicit communication between parent and the child (Cooper et al., 1993). Thus, once Chinese parents initiate and facilitate discussion with their children, children could benefit from it right away.

## Limitations

Although this study extended prior work in this area in several important ways, some limitations should be noted. One of the major limitations is the use of cross-sectional data to examine the process model. Longitudinal studies should be conducted to replicate the present findings and to examine whether changes in family income or parental education result in changes in children's academic achievement and, if so, whether parental expectation and involvement still play a mediating role in this association. Another limitation is that our parental expectation and parental involvement were not measured separately for mothers and fathers. Further research that includes both maternal and paternal reports would provide enhanced understanding of the distinct parental roles with regard to children's education and would be helpful in determining whether the differences or similarities in maternal and paternal expectation or involvement were related to children's academic outcomes. Additionally, the influence of family SES on children's reading achievement was not completely mediated by the mechanisms measured in the current study. It would be valuable for future studies to include other family variables related to family SES and children's academic achievement, such as cognitive stimulation at home, home physical environment and shared book reading with children.

## Conclusions and implications

Our findings indicated that family income and parental education are indirectly related to children's reading achievement through parental expectation and specific parental involvement behaviors. More importantly, we found that the exact nature of these links differs by gender group. These findings yield important implications for implementing family interventions and programs. First, parental expectation and specific components in parental involvement are important explanatory factors for the link between SES and achievement, which suggests that economic difficulties do not necessarily constrain child development. If parents are successful in forming accurate expectations regarding their child's performances and translating their expectations into actual behaviors of involvement in education, the negative effects of financial restrictions can be minimized. Given that Chinese parents generally already have a high educational expectation for their children (more than half of the parents expected their children to get a master's degree or higher in the present study), family intervention programs should be designed to encourage parents to discuss learning and schooling-related topics more frequently with their children or to teach parents communication skills that fit with the child's interests and needs. Second, it was found in our study that Chinese parents are deeply involved in children's education. However, only parent-child communication had a positively predictive relation with children's reading achievement, and even home monitoring played a negative role among girls, after family SES and parental expectation were controlled for. These results indicated that more involvement may not always benefit children's learning. Thus, interventions to provide valuable information regarding how to appropriately and effectively become involved in children's learning may enhance the positive effects of parental involvement on children's development outcomes. Finally, because our findings suggest that the paths linking family background variables to children's academic outcomes differed between boys and girls, intervention programs should be informed by and tailored to the characteristics and needs of the target families.

## Author contributions

Conception and design of the work was done by XG and LL. Data collection done by all authors. Analysis of data done by XG, BL, and HZ, and all other authors interpreted the data. XG, BL, and LL wrote the original draft of the manuscript, and all other authors edited and co-wrote the manuscript.

### Conflict of interest statement

The authors declare that the research was conducted in the absence of any commercial or financial relationships that could be construed as a potential conflict of interest.
